# Investigating the Autonomic Nervous System Response to Anxiety in Children with Autism Spectrum Disorders

**DOI:** 10.1371/journal.pone.0059730

**Published:** 2013-04-05

**Authors:** Azadeh Kushki, Ellen Drumm, Michele Pla Mobarak, Nadia Tanel, Annie Dupuis, Tom Chau, Evdokia Anagnostou

**Affiliations:** 1 Bloorview Research Institute, Holland Bloorview Kids Rehabilitation Hospital, Toronto, Ontario, Canada; 2 The Hospital for Sick Children, Toronto, Ontario, Canada; 3 Institute of Biomaterials and Biomedical Engineering, University of Toronto, Toronto, Ontario, Canada; 4 Department of Pediatrics, University of Toronto, Ontario, Canada; The University of Western Australia, Australia

## Abstract

Assessment of anxiety symptoms in autism spectrum disorders (ASD) is a challenging task due to the symptom overlap between the two conditions as well as the difficulties in communication and awareness of emotions in ASD. This motivates the development of a physiological marker of anxiety in ASD that is independent of language and does not require observation of overt behaviour. In this study, we investigated the feasibility of using indicators of autonomic nervous system (ANS) activity for this purpose. Specially, the objectives of the study were to 1) examine whether or not anxiety causes significant measurable changes in indicators of ANS in an ASD population, and 2) characterize the pattern of these changes in ASD. We measured three physiological indicators of the autonomic nervous system response (heart rate, electrodermal activity, and skin temperature) during a baseline (movie watching) and anxiety condition (Stroop task) in a sample of typically developing children (n = 17) and children with ASD (n = 12). The anxiety condition caused significant changes in heart rate and electrodermal activity in both groups, however, a differential pattern of response was found between the two groups. In particular, the ASD group showed elevated heart rate during both baseline and anxiety conditions. Elevated and blunted phasic electrodermal activity were found in the ASD group during baseline and anxiety conditions, respectively. Finally, the ASD group did not show the typical decrease in skin temperature in response to anxiety. These results suggest that 1) signals of the autonomic nervous system may be used as indicators of anxiety in children with ASD, and 2) ASD may be associated with an atypical autonomic response to anxiety that is most consistent with sympathetic over-arousal and parasympathetic under-arousal.

## Introduction

Autism spectrum disorders (ASDs) are a group of neuro-developmental disorders that are characterized by a triad of features: impairments in communication, deficits in social interactions, and presence of restrictive repetitive and stereotypical behaviours, interests, or activities. In addition to difficulties in these three core domains, ASDs are also associated with various co-morbidities, including psychiatric disorders. Among these, anxiety is one of the most pressing clinical concerns due to its negative impact on physical and emotional well-being [1], [2], high prevalence in this population [3], and its bidirectional relationship with the core deficits of ASD. Anxiety concerns in ASD present across all levels of functioning [4] and persist over the life span [4].

Anxiety symptoms are closely linked to the core deficits of ASD. On the one hand, core-deficits of ASD may contribute to increasing anxiety [5]. Many children with ASD are acutely aware of their social deficits [3], and experience significant anxiety related to inappropriate social behavior and expectations of social failure. Sensory deficits in understanding the external world or awareness of these difficulties [6] may also contribute to increasing anxiety. On the other hand, anxiety may drive and/or exacerbate core-deficits in ASD. For example, social anxiety may lead to further avoidance of social situations, promoting isolation from social groups [3]. In fact, anxiety has been correlated with greater degrees of loneliness and social disability [7], decreased participation in school and community [2], and negative externalizing behaviours such as aggression and self-injury [3], [8], [9]. Moreover, repetitive behaviors seen in ASD, such as hand flapping, rocking, and echolalia, may be anxiety coping mechanisms [6].

Because of the close relationship between ASD and anxiety, assessment of anxiety symptoms in children with ASD is a challenging task. Such assessment relies on self-reports or observation of overt behaviour. Self-reports are often not reliable in ASD because of deficits in communication as well as difficulties with emotional awareness and introspection [10]. Behavioural symptoms of anxiety are also difficult to recognize in ASD due to the symptom overlap between the two conditions as well as the idiosyncratic or atypical nature of anxiety symptoms in this population [10]. These difficulties necessitate the development of a physiological marker that can reliably document anxiety in ASD.

The physiological response to anxiety is orchestrated by a large network of neural structures in the central, peripheral, and endocrine systems. The peripheral nervous system holds special promise for the development of an anxiety marker as the response of this system can be measured non-invasively and with relative ease. In particular, the autonomic branch of the peripheral nervous system is activated during the anxiety response to mobilize appropriate behavioural responses to stressful stimuli [11]. The ANS response to stress, known as the fight or flight response, generally involves the activation and inhibition of the sympathetic and parasympathetic branches of the ANS, respectively. This stress response results in several physiological changes that can be measured non-invasively. These include:

- Changes in cardiac activity: The sympathetic and parasympathetic branches of the ANS have excitatory and inhibitory effects on cardiac function, respectively. Specifically, activation of the sympathetic system during stress generally increases heart rate and cardiac contractibility [12].
- Changes in perspiration: Eccrine sweat glands have predominantly sympathetic cholinergic innervations [13] and their activity increases when the sympathetic nervous system responds to stress. In contrast to thermoregulatory sweating which occurs over the entire body, this type of ``emotional sweating'' generally occurs in the palms of the hands, as well as the axillae and soles of the feet [14]. Because the amount perspiration affects the electrical conductivity of the skin, sympathetically-induced changes in perspiration can be measured non-invasively as electrodermal activity (EDA). A high correlation between the level of sympathetic activity and phasic changes in EDA has been reported [13].
- Changes in skin temperature: Arousal of the sympathetic nervous system generally results in vasoconstriction, roughly proportional to the level of neural activity [15]. Furthermore, cutaneous micro-circulation also affects skin temperature. Because arterioles of the fingertip skin have sympathetic, adrenergic constrictor nerves, sympathetically-induced vasoconstriction can be measured indirectly by observing transient changes in fingertip temperature [16].

Using the above measures, several studies have investigated the physiological patterns associated with behaviours seen in ASD. For example, children with ASD were found to have a dampened EDA response to faces as compared to objects, and self-stimulation activities seemed to calm the ANS [17]. In another study, children with ASD showed a stronger EDA response to another person's gaze than typically developing children [18], suggesting that over-arousal to eye contact may contribute to the atypical gaze patterns seen in ASD. A blunted parasympathetic response during mental tasks has also been reported in ASD [19].

The above evidence supports the feasibility of using the ANS response as a physiological marker of atypical behaviours in ASD. Despite this, however, little is known about the ANS response to anxiety in ASD. This response has been investigated in a handful of studies which have produced inconsistent results. In particular, decreased heart rate response to stress was reported in children and adults with ASD in a public speaking task [20], [21]. The differences were attributed either to increased parasympathetic activity or to the down-regulation of the ANS due to a chronic state of arousal. Consistent with the ANS hyper-arousal hypothesis, another study [22] reported higher basal levels of heart rate and reduced variance in responsivity to different social and non-social stressors in children and youth with ASD as compared to typically developing individuals. A third study, however, did not find significant differences in EDA and vagal tone responses to the Trier Social Stress Test (oral story, serial subtractions, tracing a star through a mirror, debriefing) between individuals with ASD and typically developing controls [23]. This study also found that unlike the control group, the ASD group demonstrated a blunted salivary cortisol response following the stressor, which may be explained by chronic stress. The discrepancies in the above findings may be related to differences in the levels of anxiety induced by the various tasks in the participant groups [20]. Additionally, differences in other cognitive and performance variables (e.g., IQ, age, and task performance) may contribute to the discrepant results of these studies.

Given the limited understanding of the ANS anxiety response in ASD, the goal of this study was to further characterize this response. To gain a more comprehensive picture of ANS function, three indices of autonomic arousal were used (heart rate, electrodermal activity, and skin temperature). Two research questions were specifically investigated:

1. Can physiological responses to anxiety be detected in children with ASD?
2. Are the patterns of physiological responses to anxiety atypical in children with ASD?

To address the challenges involved in interpreting the results of previous studies, additional measures of state and trait anxiety, as well as measures of cognitive functioning and task performance were employed.

## Methods

### Ethics Statement

The Bloorview Research Institute research ethics board approved the study. Written consent was obtained from all participants who were deemed to have the capacity for consent. For all other participants, assent and written consent were obtained from the children and their legal guardians, respectively.

### Participants

We recruited a sample of typically developing children (n = 18) and children with ASD (n = 15) for the study. None of the participants had a history of a diagnosed neurological or psychiatric disorder, was on medications that affect ANS function, or was born prior to 35 weeks gestational age.

Typically developing children were recruited from children of staff at Holland Bloorview Kids Rehabilitation Hospital in Toronto, Canada and through advertising at nearby schools. Children with ASD were recruited through the Autism Research Centre at Holland Bloorview Kids Rehabilitation Hospital. All children in the ASD group were by expert clinical team, using DSM-IV criteria supported by the Autism Diagnostic Observation Schedule [24].

### Task Design

Each participant completed an anxiety-inducing task, preceded and followed by a baseline task. For the baseline tasks, participants watched a movie of their choice for 30 minutes. Given the difficulties of task transition in the ASD group, all participants were provided with verbal prompts when there were 10 and 5 minutes left of the movie-watching activity.

**Figure 1:**
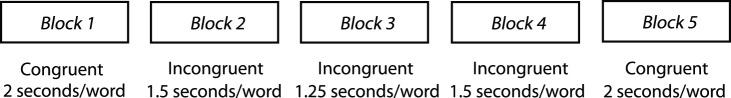
Structure of the Stroop Color-Word Interference Task. The duration of each block is one minute.

For the anxiety-inducing task, participants completed a computerized version of the Color Stroop (Color-Word Interference) Task [25], commonly used to elicit stress reactions in studies of autonomic nervous system function [26], [27]. The task involves presentation of words corresponding to color names, printed in differently colored letters. The participants were required to name the color of the letters while ignoring the printed word. The Stroop task in this study consisted of 5 blocks, each one minute long. As shown in Figure 1, the stimulus presentation frequency was varied from 2 to 1.25 seconds/word over the blocks.

After the Stroop task was explained to the participants, they were asked to demonstrate their understanding of the task with ten practice words. All participant completed this practice task without errors.

### Measures of Behaviour and Cognitive Function

Intellectual functioning was measured by the Stanford-Binet Intelligence Scale. For two of the children in the ASD group, existing intelligence scores (Wechsler Intelligence Scale) were used.

To further characterize the study sample in terms of trait anxiety, the generalized anxiety symptom severity scores from the Child and Adolescent Symptom Inventories were used (child version was used for children 12 or younger and the adolescent version was used otherwise). Participants also completed the State-Trait Anxiety Inventory (STAI) as a self-reported measure of state anxiety. The STAI was completed before and after each baseline.

### Physiological Measurements

We measured blood volume pulse (BVP), electrodermal activity (EDA), and skin temperature signals using FDA-approved sensors from Flexcomp Infiniti, Thought Technology Ltd. Sensors were attached to the non-dominant hand using breathable tape (for BVP and skin temperature) and velcro straps (for EDA).

To measure BVP, a photoplethysmography [28] sensor was attached to the palmar surface of the distal phalanges of the first digit of the hand. EDA was measured as skin conductance using a pair of 10 mm diameter dry Ag-AgCl electrodes secured to the palmar surface of the proximal phalanges of the second and third digits of the non-dominant hand. Skin temperature was measured using a thermistor fastened to the palmar surface of the distal phalanx of the fourth digit of the hand.

All signals were sampled at a frequency of 256 Hz and recorded to a laptop for subsequent off-line analyses.

### Data Analysis

The BVP, EDA, and skin temperature signals were low-pass filtered (Butterworth filter, cut-off frequency of 10 Hz). EDA and skin temperature signals were then detrended over the entire study session to eliminate the effects of acclimation and thermoregulation. For the baseline periods, the first and last 10 minutes of the task were discarded to account for acclimatization and the effects of anticipation anxiety due to examiner prompts, respectively.

For BVP, the mean value of the heart rate over the baselines and the task period were used for the analyses. Heart rate was computed as the inverse of the pulse-to-pulse intervals, which were obtained using the minima of the BVP signal. For EDA, the mean signal value and the number of electrodermal reactions (EDR) were extracted. An EDR was defined as peaks in the EDA waveform with a minimum height of 0.05 microsiemens and interpeak distance of 1 second. The interpeak distance was chosen to correspond to the minimum stimulus separation of 1 word per second to capture specific electrodetermal reactions due to the stimulus [13]. For skin temperature, the mean value was extracted over the baseline and task periods.

Statistical analyses were performed using SAS version 9.3 (SAS Institute, Cary, NC). Stepwise regression, combining forward selection and backward elimination, was used to determine which of the demographic variables (age, IQ, gender) significantly affected the generalized anxiety scores and Stroop task performance. Repeated measures multiple linear regression analysis was used to investigate the effect of group (ASD versus TD) and condition (movie 1, Stroop task, movie 2) on the STAI scores while controlling for demographic variables (gender, IQ, age). To answer the two research questions, repeated measures multiple regression analysis was employed. Specifically, we examined the significance of group (ASD versus TD) and condition (movie 1, Stroop task, movie 2) on mean heart rate, mean EDA level, number of EDA reactions, and mean skin temperature using separate repeated measures models. Linear regression was used for mean heart rate, mean EDA, and mean skin temperature, whereas poisson regression was employed for the number of EDRs. Gender, IQ, age, task performance, and generalized anxiety scores were included as covariates in these models. Because EDA and skin temperature are known to be affected by overall skin temperature, we also included the mean temperature over the session as a covariate in the model.

## Results

### Participant Characteristics

One typically developing child and three children with ASD did not complete the study and were excluded from the analyses. The final sample consisted of 17 typically developing children and 12 children with ASD (6 and 6 with diagnoses of Asperger's and autism, respectively). Participant characteristics are reported in Table 1. The two groups differed significantly on full scale IQ (*p* = 0.003), but not on age or gender.

**Table 1:** Participant characteristics.

	TD	ASD	p value
n	17	12	
Age	10.9 ± 2.3 (range 8–15)	11.3 ± 2.3 (range 8–15)	t(24.2) = −0.4, p = 0.7
Full scale IQ	108.7 ± 9.2	88.1 ± 23.0	t(13.5) = 2.9, p = 0.01
Gender (male:female)	11∶6	10∶2	Fisher's exact test, p = 0.4

### Behavioural Measures

Stepwise linear regression revealed that group and IQ best predicted the generalized anxiety scores obtained from the Child and Adolescent Symptom Inventories (model *R^2^* = 0.49, group partial *R^2^* = 0.36, IQ partial *R^2^* = 0.13). The generalized anxiety scores were significantly higher for the ASD group than the TD group (ASD: 7.2 ± 0.9, TD: 1.3 ± 0.7; t(25) = −4.9, p<0.0001). IQ was positively associated with the severity of anxiety symptoms (regression coefficient = 0.08, t(25) = 2.46, p = 0.02). The group x IQ interaction was not significant (F(1,24) = 0.5,p = 0.48).

**Figure 2:**
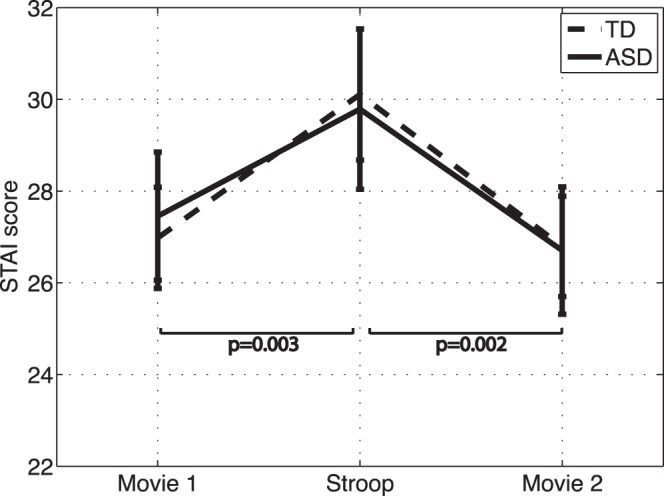
STAI scores during the movie and Stroop task periods. Bars represent standard error.

- Group: Figure 2 shows the STAI scores before and after the Stroop task for the two groups. Repeated measures analysis did not show a significant group x condition interaction (F(2,23) = 0.2, p = 0.8).
- Condition: Post-hoc analysis revealed a significant difference between scores for the two movie-watching periods and the Stroop task (movie 1 and Stroop: −2.72 ± 0.81 (t(26) = −3.3, p = 0.003), Stroop and movie 2∶3.2 ± 0.9, t(26) = 3.51, p = 0.002).

### Performance on Stroop task

Using the demographic variables (age, gender, IQ) and the generalized anxiety scores, stepwise linear regression revealed that group and age best predicted the Stroop task performance measured as the percentage of correctly named colors (model *R^2^* = 0.65, group partial *R^2^* = 0.33, age partial *R^2^* = 0.32). Typically developing children performed more accurately than the ASD group (TD: 90.0% ± 2.0, ASD: 76.7% ± 2.4; F(1,26) = 18.4, p = 0.0002). Task performance was positively associated with age (regression coefficient = 3.33, t(26) = 4.86, p<0.0001). The interaction of group x age was not significant (F(1,25) = 0.02, p = 0.89).

### Physiological Measures

#### Heart rate

**Figure 3:**
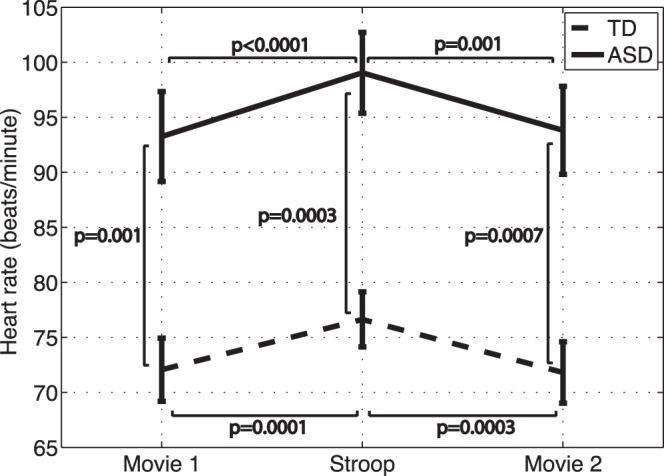
Mean heart rate during the movie watching and Stroop task periods. Bars represent standard error.

Mean heart rate values for the two groups are depicted in Figure 3 for the movie-watching and Stroop tasks.

- Group: Repeated measures analysis did not reveal a significant group x condition interaction (F(2,21) = 0.5, p = 0.6) after controlling for age, IQ, gender, Stroop test performance, and generalized anxiety score. Excluding IQ, generalized anxiety, and task performance from the model did not alter the findings.
- Post-hoc analyses showed that the ASD group had significantly higher heart rate than the TD group for the all three tasks (first movie difference: −21.2 ± 5.6, t(21) = −3.8, p = 0.001; Stroop difference: −22.4 ± 5.2, t(21) = −4.3, p = 0.0003; second movie difference −22.0 ± 5.5, t(21) = −4.0, p = 0.0007).
- Condition: Heart rate during the Stroop test was significantly higher than the two movie-watching periods for both the TD (movie 1 and Stroop difference: −4.6 ± 1.0, t(21) = −4.7, p = 0.0001; movie 2 and Stroop difference: 4.8 ± 1.1, t(21) = 4.4, p = 0.0003) and ASD groups (movie 1 and Stroop difference: −5.8 ± 1.2, t(21) = −4.8, p<0.0001; movie 2 and Stroop difference: 5.2 ± 1.4, t(21) = 3.8, p = 0.001). Mean heart rate was not significantly different between the two movie-watching periods for either group (t(21)< −0.6, p>0.5).

#### Electrodermal activity

**Figure 4:**
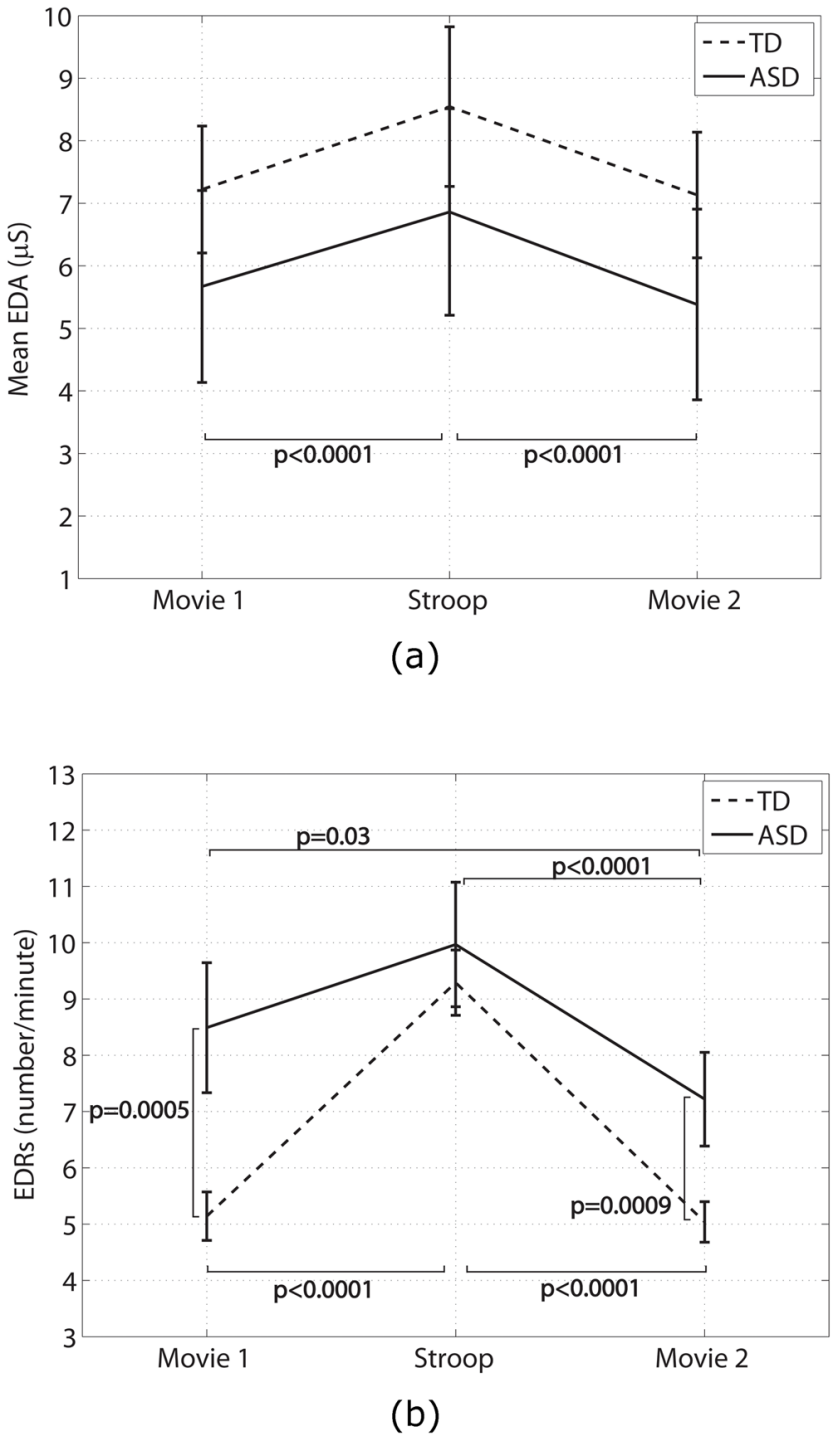
Electrodermal activity before and after the Stroop task: (a) mean EDA, (b) EDR. Bars represent standard error.

Mean EDA and number of EDRs are shown in Figure 4 for the movie-watching and Stroop test periods.

- Group: Repeated measures analysis revealed a significant group x condition interaction only for the number of EDRs (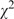
(2) = 7.7, p = 0.02) after controlling for age, IQ, gender, Stroop test performance, and generalized anxiety score. Excluding IQ, generalized anxiety, and task performance from the model did not alter the findings.
- Post-hoc analysis revealed that the ASD group had a significantly higher number of EDRs than the TD group during the two movie-watching periods (first movie difference (log): −0.5 ± 0.14, z = −3.5, p = 0.0005; second movie difference (log): −0.4 ± 0.11, z = −3.3, p = 0.0009). However, the number of EDRs was not significantly different between the group for the Stroop task (difference (log): −0.1 ± 0.09, z = −0.8, p = 0.5).
- Condition: Mean EDA was significantly higher during the Stroop test than the two movie-watching periods (movie 1 and Stroop difference: −1.3 ± 0.2, t(20) = −6.8, p<0.0001; movie 2 and Stroop difference: 1.4 ± 0.2, t(20) = 7.2, p<0.0001). However, mean EDA did not differ significantly between the two movie-watching periods (movie 1 and movie 2 difference: 0.2 ± 0.1, t(20) = 1.7, p = 0.1).

For the TD group, the number of EDRs during the Stroop test was significantly higher than the two movie-watching periods (movie 1 and Stroop difference (log): −0.6 ± 0.06, z = −9.9, p<0.0001; movie 2 and Stroop difference (log): 0.6 ± 0.06, z = 10.0, p<0.0001), but there was no significant difference in the number of EDRs between the two movie-watching periods (z = 0.3, p = 0.8). For the ASD group, however, the number of EDRs was only significantly different between the Stroop test and the second movie-watching periods, and the two movie-watching periods (movie 1 and Stroop difference (log): −0.2 ± 0.1, z = −1.4, p = 0.2; movie 2 and Stroop difference (log): 0.3 ± 0.07, z = 4.4, p<0.0001; movie 1 and movie 2 difference: 0.2 ± 0.07, z = 2.2, p = 0.03).

#### Skin Temperature

**Figure 5:**
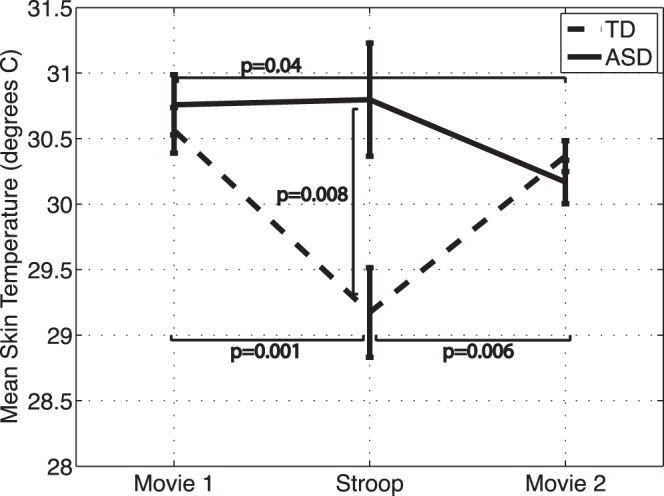
Mean skin temperature during the movie watching and Stroop task periods. Bars represent standard error.

The mean skin temperature for the Stroop task and the two movie-watching periods are shown in Figure 5 for the TD and ASD groups.

- Group: Repeated measures analysis revealed a significant group x condition interaction (F(2,20) = 4.29, p = 0.03) after controlling for age, IQ, gender, Stroop test performance, and generalized anxiety score. Excluding IQ, generalized anxiety, and/or task performance from the model did not alter the findings.

Post-hoc analysis revealed that the ASD group had significantly higher mean skin temperature than the TD group during the Stroop task, but not during the movie periods (first movie difference: −0.20 ± 0.3, t(20) = −0.66, p = 0.52; Stroop difference: −1.6 ± 0.6, t(20) = −2.9, p = 0.008; second movie difference: 0.19 ± 0.2, t(20) = 0.87, p = 0.4).

- Condition: Mean temperature was significantly lower during the Stroop task than the two movie-watching periods for the TD group (movie 1 and Stroop difference: 1.4 ± 0.4, t(20) = 3.81, p = 0.001; movie 2 and Stroop difference: −1.2 ± 0.4, t(20) = −3.1, p = 0.006, movie 1 and movie 2 difference: 0.20 ± 0.2, t(20) = 0.9, p = 0.38). For the ASD group, only the difference between the two movie-watching periods was significance (movie 1 and Stroop difference: −0.04 ± 0.5, t(20) = −0.09, p = 0.9; movie 2 and Stroop difference: 0.62 ± 0.5, t(20) = 0.3, p = 0.2, movie 1 and movie 2 difference: 0.60 ± 0.3, t(20) = 2.16, p = 0.04).

## Discussion

### 

#### Severity of anxiety symptoms

Anxiety symptoms are reported to be more prevalent in ASD than in the typically developing population, as well as in populations with other developmental disorders, such as specific language impairment [6] and intellectual disability [29]. Consistent with these reports, the ASD group in our study had more severe generalized anxiety disorder symptoms than the TD group.

We also found a positive association between the severity of anxiety symptoms and IQ, an observation reported in existing literature [30]. One explanation for this association may be that children with higher IQ may have have greater awareness of their deficits and negative life events, and therefore experience higher levels of anxiety. Another explanation may be that the children with higher IQs may have a greater ability to express typically recognized signs of anxiety.

### Performance on the Stroop Task

Previous literature has generally reported intact performance on the Color Stroop task for children with ASD [31]–[33]. However, in this study we found that the ASD group performed worse than the typically developing group on the Stroop task. This apparent discrepancy may be due to the timed nature of the Stroop task used in the present study which may have increased the processing demands of this task.

### Self-report Measure of Anxiety

The self-report (STAI) and physiological measures confirmed that the Stroop task successfully elicited a stress-response in both the ASD and TD groups. The STAI results further indicated that the levels of perceived anxiety did not differ significantly between the groups. This result is encouraging as differences in the perceived level of stress have been suggested to affect findings of previous studies of physiological function in children with ASD. Caution must be taken in interpreting this finding given the known deficits in introspection, emotion awareness, and communication of emotions in ASD [10].

### Physiological Measures

The anxiety condition (Stroop task) caused significant increases in heart rate and tonic levels of EDA in both groups, a pattern consistent with the expected ANS arousal in response to stress. This further confirms that the anxiety condition did in fact elicit an anxiety response in both groups.

Our results also suggest that state anxiety may be detected based on measurement of ANS signals, further supporting the feasibility of using these physiological signals as language-free and objective indicators of anxiety in children with ASD. This is important as recognition of anxiety symptoms in ASD, especially in lower-functioning individuals, is a challenging task. The challenge relates to the symptom overlap between ASD and anxiety disorders (e.g., panic attacks and obsessions), idiosyncratic or atypical anxiety symptoms, and limited understanding of subjective experiences of anxiety due to deficits with communication and introspection [10]. The difficulties in recognition of anxiety symptoms can lead to diagnostic overshadowing [10] and complicate treatment of anxiety in ASD. A physiological measure of anxiety can partially overcome these difficulties and complement existing assessment methods.

Although our results support the feasibility of physiological measures of anxiety in children with ASD, the findings also suggest that the pattern of ANS response to anxiety may be atypical in this population. The following sections discuss the findings related to each of the signals examined in the present study.

#### Group differences in heart rate

The ASD group had elevated heart rate compared to the TD group for the movie-watching and Stroop tasks. The cardiac response is determined by the inhibitory and excitatory effects of the parasympathetic and sympathetic branches of the ANS, respectively. Therefore, the finding of elevated heart rate may indicate the under-arousal of the parasympathetic system and/or over-arousal of sympathetic nervous system. Reduced parasympathetic tone and increased sympathetic tone during rest [34], a blunted parasympathetic response during a mental task (mental arithmetic) [19], and lowered levels of respiratory sinus arrhythmia (measure of parasympathetic activity) [35], [36] have been previously reported in ASD. This evidence collectively suggests decreased parasympathetic activity in ASD, which can lead to an over-aroused cardiac ANS response.

Many central and peripheral structures may be implicated in producing an atypical cardiac ANS response. At the central level, the amygdala plays an important part in controlling the autonomic arousal processes, including changes in heart rate [37], [38]. The amygdala activates during threat and uncertainty and contributes to the arousal of the autonomic nervous system through its projections to the hypothalamus and the brain stem. As such, the amygdala may contribute to the atypical modulation of the ANS response. In fact, ASD is associated with structural and functional differences in the amygdala (e.g., overgrowth in volume early in life [39], as well as atypical activations during social and emotional tasks [11]). Moreover, these differences have also been associated with anxiety symptom severity in ASD [40], [41]. Further investigation is needed to study the relationship between atypical findings in the amygdala and ANS over-arousal.

The central and peripheral nervous systems are connected through thoracolumbar innervations and cranial nerves. Given the reports of decreased parasympathetic tone in ASD, potential dysfunction of cranial nerve X, or the vagus could not be ruled out. The vagus exerts parasympathetic control over the heart and its dysfunction has been linked to difficulties with various social engagement functions, including those associated with ASD [42].

#### Group differences in EDA

The EDA response patterns provide further insight into the activity of the sympathetic nervous system as the palmar eccrine sweat glands are largely sympathetically innervated. In this study, we found that the number of EDRs during the first movie watching period was higher than typical for the ASD group. Moreover, unlike the TD group, the number of EDRs did not increase during the Stroop task for the ASD group. This pattern is consistent with the hyper-arousal of the sympathetic nervous system resulting in decreased responsivity to stress.

We did not find any significant differences between the ASD and TD groups in the mean EDA level (reflects tonic sympathetic activity). It is argued that tonic EDA is an indicator of general states of arousal whereas phasic EDRs are useful for studying differences related to behavioural and psychopathological states [13]. As such, the mean EDA measure may not be sensitive to differences between the two groups studied herein.

There is evidence to suggest that the production of the EDRs due to affective processes is centrally mediated by the limbic structures, including the anterior cingulate cortex (ACC) and the amygdala [13], [14]. As previously mentioned, ASD is associated with structural and functional differences in the amygdala. Atypical findings in the ACC have also been reported in the ASD. These include decreased volume and diminished activation in the ACC [43]. These regions may therefore be interesting targets for future investigations of ANS function in ASD.

#### Group differences in skin temperature

Sympathetic stimuli are typically expected to induce vasoconstriction in arterioles of the fingertip skin, leading to a decrease in skin temperature [16]. In this study, we did not find significant differences in skin temperature between the ASD and TD groups during the movie-watching periods. However, unlike the TD group who exhibited a decrease in skin temperature during anxiety, skin temperature did not change significantly in the ASD group. As was the case with the EDR response patterns, this finding may be explained by chronic over-arousal of the sympathetic nervous system (vasoconstriction), resulting in decreased responsivity of the ANS.

Although there are no direct studies of vasoconstriction mechanisms in ASD, increased vasoconstriction due to hyper-arousal of the sympathetic system has been suggested previously as an explanation for elevated resting heart rate, diastolic blood pressure, and mean arterial pressure observed in ASD [34]. Another explanation for our results may be the potential pathology of the adrenergic constrictor nerves that hamper vasoconstrictor mechanisms in ASD. Further investigation is needed to explore this possibility.

### Evidence Supporting Hyper-Arousal of the ANS in ASD

In this study, we reported atypical findings related to heart rate, EDA, and skin temperature in ASD. Overall, simultaneous examination of our three measurement modalities implicate both branches of the ANS to the atypical anxiety response in ASD. As previously mentioned, heart rate is modulated by the both the sympathetic and parasympathetic branches of the ANS whereas EDA and skin temperature are regulated by the sympathetic branch alone. Our findings related to EDA and skin temperature are most consistent with the hyper-arousal of sympathetic branch of the nervous system in ASD, leading to decreased responsivity to anxiety-inducing stimuli.

If our atypical findings were related to the sympathetic system alone, however, we would expect the heart rate pattern to mirror that of EDA and skin temperature [44]. This was not the case in our study where we found an elevated heart rate during baseline and anxiety conditions in the ASD group, but did not find an atypical heart rate response to anxiety (i.e., heart rate increase appropriately during anxiety). The disparity between the heart rate, and EDA and skin temperature suggests the contribution of both branches of the ANS to our atypical findings. In particular, this pattern may suggest a blunted parasympathetic response to anxiety given the decreased reactivity of the sympathetic branch to this condition. Moreover, the finding of elevated heart rate during baseline is consistent with overall decreased parasympathetic tone in ASD, which has been previously reported [34]. Interestingly, our findings of elevated heart rate mirror the reports of increased tonic pupil size in ASD [45]. In particular, pupil diameter is regulated by dilator and sphincter muscles whose activity is in turn influenced by the sympathetic and parasympathetic systems, respectively (increased sympathetic activity and/or decreased parasympathetic activity lead to pupil dilation) [44]. Thus, the increased pupil diameter in ASD may be attributed to increased sympathetic activity or blunted parasympathetic activity.

Overall, our findings in this study and the previous reports of atypical activity in the cardiac and pupillary systems, collectively suggests over-arousal of the ANS related to increased sympathetic and decreased parasympathetic tone in ASD.

### Study Limitations

Our results suggest that ANS signals can be used to detect anxiety in children with ASD, at least in the experimental setting used in this study. However, it is known that ANS signals also change with cognitive load and other mental processes such as attention and response inhibition. Because the Stroop task also invokes these processes, future work is needed to examine the specificity of the ANS indicators with respect to other cognitive operations. Moreover, while our results showed an atypical pattern of ANS activation in ASD, we did not examine whether or not this atypical pattern is unique to ASD.

The small sample size used in the present study may have limited our ability to detect significant group differences in the physiological measures. This small sample also hindered the analysis of subgroups that may exhibit different physiological responses to anxiety (over-arousal versus under-arousal), as well as the influence of covariates, such as IQ, which may have influenced the physiological changes. As this was a pilot study, we have limited data on past interventions and current characterization of core symptom domains. Both features should be explored in relation to our findings in the future.

The present study examined group differences in physiological measures averaged over the entire task interval. An interesting direction for future studies would be to investigate the presence of a delayed response in the ASD group by examining the physiological indicators over shorter time scales that correspond to the expected response times of the ANS signals.

Finally, this study examined ANS function by measuring physiological responses that result from complex interactions of several structures in the central, peripheral, and the endocrine systems. Our results suggest an atypical ANS response to anxiety, however, future studies are needed to fully understand the mechanisms underlying these differences.

### Conclusion

The results of this study generally support the feasibility of using a physiological marker of anxiety in children with ASD based on patterns of ANS function, though future work is needed to document this out of an experimental setting. The findings also suggest that an atypical ANS response to anxiety, most consistent with over-arousal, may be associated with ASD. Future studies are needed to pinpoint the underlying mechanisms in the central and peripheral nervous systems that may contribute to this atypical response.
